# An apoptosis-inducing factor controls programmed cell death and laccase expression during fungal interactions

**DOI:** 10.1007/s00253-023-12988-1

**Published:** 2024-01-13

**Authors:** Junnan Fang, Gang Zhou, Huifang Zhao, Dengdeng Xie, Jingna Zhang, Ursula Kües, Yazhong Xiao, Zemin Fang, Juanjuan Liu

**Affiliations:** 1 School of Life Sciences, Anhui University, Hefei, 230601 Anhui China; 2 Anhui Key Laboratory of Modern Biomanufacturing, Hefei, 230601 Anhui China; 3 Anhui Provincial Engineering Technology Research Center of Microorganisms and Biocatalysis, Hefei, 230601 Anhui China; 4 Molecular Wood Biotechnology and Technical Mycology, Büsgen‑Institute, University of Goettingen, Büsgenweg 2, 37077 Goettingen, Germany

**Keywords:** Interspecific interaction, Apoptosis, Apoptosis-inducing factor, Reactive oxygen species, Laccase

## Abstract

**Abstract:**

Apoptotic-like programmed cell death (PCD) is one of the main strategies for fungi to resist environmental stresses and maintain homeostasis. The apoptosis-inducing factor (AIF) has been shown in different fungi to trigger PCD through upregulating reactive oxygen species (ROS). This study identified a mitochondrial localized AIF homolog, *Cc*AIF1, from *Coprinopsis cinerea* monokaryon Okayama 7. Heterologous overexpression of *Cc*AIF1 in *Saccharomyces cerevisiae* caused apoptotic-like PCD of the yeast cells. *Ccaif1* was increased in transcription when *C. cinerea* interacted with *Gongronella* sp. w5, accompanied by typical apoptotic-like PCD in *C. cinerea*, including phosphatidylserine externalization and DNA fragmentation. Decreased mycelial ROS levels were observed in *Ccaif1* silenced *C*. *cinerea* transformants during cocultivation, as well as reduction of the apoptotic levels, mycelial growth, and asexual sporulation. By comparison, *Ccaif1* overexpression led to the opposite phenotypes. Moreover, the transcription and expression levels of laccase Lcc9 decreased by *Ccaif1* silencing but increased firmly in *Ccaif1* overexpression *C*. *cinerea* transformants in coculture. Thus, in conjunction with our previous report that intracellular ROS act as signal molecules to stimulate defense responses, we conclude that *Cc*AIF1 is a regulator of ROS to promote apoptotic-like PCD and laccase expression in fungal-fungal interactions. In an axenic culture of *C. cinerea*, *Cc*AIF1 overexpression and H_2_O_2_ stimulation together increased laccase secretion with multiplied production yield. The expression of two other normally silent isozymes, Lcc8 and Lcc13, was unexpectedly triggered along with Lcc9.

**Key points:**

*• Mitochondrial CcAIF1 induces PCD during fungal-fungal interactions*

*• CcAIF1 is a regulator of ROS to trigger the expression of Lcc9 for defense*

*• CcAIF1 overexpression and H*_*2*_ *O*_*2*_ *stimulation dramatically increase laccase production*

**Supplementary Information:**

The online version contains supplementary material available at 10.1007/s00253-023-12988-1.

## Introduction

Apoptosis, one type of programmed cell death (PCD), represents one of the highly conserved cellular eukaryotic suicide programs triggered by extrinsic or intrinsic cellular stimuli. It has been described and investigated in detail in mammalian cells (Hamann et al. 2008; Rico-Ramírez et al. 2022). In fungi, apoptosis is called apoptotic-like PCD because their manifestation of PCD differs from that of mammals (Shlezinger et al. 2012; Hardwick 2018). Apoptotic-like PCD is involved in various biological processes such as stress adaptation, development, aging, and host-pathogen interactions in fungi (Hamann et al. 2008; Häcker 2018; Gonçalves et al. 2020). During fungal-fungal antagonistic interaction, compounds secreted by a competitor may trigger apoptotic-like PCD in a fungus, providing a remarkable selection advantage for nutrients (Shlezinger et al. 2012). At the same time, apoptotic removal of damaged cells could also contribute to a fitter, better-adapted population in the long term (Hamann et al. 2008; Saladi et al. 2020). Thus, apoptotic-like PCD is an important strategy for fungi to gain advantage in antagonistic fungal competition. However, the mechanisms driving apoptotic-like PCD during fungal antagonistic interactions remain unclear.

Apoptosis-inducing factor (AIF) is a conserved flavoprotein among eukaryotic kingdoms (Novo et al. 2021). It is a caspase-independent apoptosis effector located in the mitochondrial intermembrane space (Miramar et al. 2001; Elguindy and Nakamaru-Ogiso 2015). Upon proteolytic induction, AIF translocates from mitochondria to the nucleus, leading to several hallmarks of apoptosis, such as chromatin condensation and DNA degradation (Delavallée et al. 2011; Cho et al. 2018). The cytoplasmic AIF-homologous mitochondrion-associated inducer of death (AMID) regulates apoptotic-like PCD in a similar way. Many fungal AIF or AMID homologs have been characterized. For example, in an *AIF* (*Ynr074cp*) knockout strain of baker’s yeast *Saccharomyces cerevisiae*, H_2_O_2_- and acetic acid–induced apoptotic-like PCD is significantly attenuated (Wissing et al. 2004). Aif1 is also required for apoptotic-like PCD in the basidiomycetous yeast *Cryptococcus neoformans*. Its deletion promotes chromosome aneuploidy and fluconazole resistance (Semighini et al. 2011). In contrast, AIF1 from the ascomycete yeast *Candida albicans* plays a dual role in regulating cell death under different concentrations of stress-causing agents. AIF1 deletion leads to attenuated apoptotic-like PCD under 2 mM H_2_O_2_ or 20 mM acetic acid but results in reversed sensitivity when treated with more severe stresses (Ma et al. 2016). In contrast to these unicellular yeast species, filamentous fungal species typically have several AIF or AMID paralogs. According to reports in *Podospora anserina* (Brust et al. 2010), *Neurospora crassa* (Carneiro et al. 2012), and *Aspergillus nidulans* (Savoldi et al. 2008; Dinamarco et al. 2010), at least some of them play a role in apoptotic-like PCD during stress. Accordingly, AIF or AMID-related PCD is speculated to be universal and critical among all fungi. No AIF or AMID homologs have been identified and characterized in multicellular basidiomycetes. Cytological studies reported that PCD in basidiomycetes is related to heteroincompatibility reactions of mycelia of *Helicobasidium mompa* and *Rosellinia necatrix* (Inoue et al. 2011a, 2011b), mycelial secondary metabolite (ganoderic acid) production in *Ganoderma lucidum* (Zhu et al. 2019), mycelial aging in *Lentinula edodes* (Gao et al. 2019), targeted tissue degradation in fruiting body development for shaping the mushrooms in *Coprinopsis cinerea* and *Agaricus bisporus* (Lu and Sakaguchi 1991; Umar and Van Griensven 1997), checkpoints in the progression of meiosis in *C. cinerea* (Celerin et al. 2000; Lu et al. 2003; Sugawara et al. 2009), and its assorciation with heat stress in *Pleurotus* species (Song et al. 2014).

AIF and AMID share FAD-binding motifs in their N-termini. Independent of their apoptogenic function, they also possess NAD(P)H oxidoreductase activities capable of generating superoxide radicals (Urbano et al. 2005; Joza et al. 2009; Elguindy and Nakamaru-Ogiso 2015; Herrmann and Riemer 2021). Under normal conditions, AIF and AMID participate in respiratory complex I assembly, play essential roles in oxidative phosphorylation and redox control, and contribute to regulating reactive oxygen species (ROS) (Joza et al. 2009). ROS, comprising both free radical oxygen intermediates and non-free ones, including ∙O_2_, OH^−^, and H_2_O_2_, possess many physiological functions in fungi, including signal transduction, interspecific interactions, and secondary metabolite synthesis (Miranda et al. 2013; Breitenbach et al. 2015; Holze et al. 2018; Liu et al. 2022). Research has shown that enhanced ROS formation is a prerequisite for AIF to be carbonylated, proteolytically cleaved, and released from mitochondria (Norberg et al. 2010; Su et al. 2021). In *S. cerevisiae* (Li et al. 2006) and *C. albicans* (Ma et al. 2016), AIF or AMID deficiency leads to decreased ROS production under low levels of oxidative stress but it results in higher ROS levels when exposed to higher concentrations of H_2_O_2_, indicating a complicated link between ROS and AIF/AMID.

Previously, we reported that intracellular ROS acted as signal molecules to stimulate defense responses in *C. cinerea* by expressing various detoxification proteins, including laccase Lcc9, during interaction with the mucoromycete *Gongronella* sp. w5 (Liu et al. 2022). In this study, based on gene silencing and overexpression analysis, we describe an AIF homolog *Cc*AIF1 in *C. cinerea* monokaryon Okayama 7 as a regulator of ROS to promote Lcc9 expression. *Ccaif1* induced apoptotic-like PCD in *C. cinerea* cells grown in cocultures with *Gongronella* sp. w5, but *Ccaif1* silencing disrupted this process and slowed *C. cinerea* mycelial growth and asexual sporulation, as well as Lcc9 expression. Thus, AIF-related PCD is an effective defense mechanism for *C. cinerea* in fungal-fungal interactions. Furthermore, based on the mechanisms we elucidated, a new strategy for the highest enzyme yields was established by combining *Cc*AIF1 overexpression and H_2_O_2_ stimulation to trigger *C. cinerea* laccase production in an axenic culture.

## Materials and methods

### Fungi and culture media

*C. cinerea* Okayama 7 (#130; *A43*, *B43*, *ade8*) (ATCC No. MYA-4618™) and *Gongronella* sp. w5 (China Center for Type Culture Collection No. AF2012004) were maintained on YMG agar (yeast malt glucose; per liter, 4 g yeast extract, 10 g malt extract, 4 g glucose, and 15 g agar) or PDA (potato dextrose agar; per liter, filtrate of 200 g boiled- potato, 20 g glucose, and 15 g agar) plates at 4 °C according to Pan et al. (2014). All the fungal cultivation experiments were conducted at 37 °C.

### Axenic culture and separated coculture

To maintain normal growth, axenic cultivation of *C. cinerea* in liquid FAHX medium (Fructose DL-asparagine HX; per liter, 15.0 g fructose, 1.5 g DL-asparagine, 1.0 g KH_2_PO_4_, 0.5 g MgSO_4_·7H_2_O, 0.1 g Na_2_HPO_4_·5H_2_O, 10.0 g CaCl_2_, 1.0 mg FeSO_4_·7H_2_O, 28.0 mg adenine, 2.0 mg CuSO_4_·5H_2_O, and 50.0 μg vitamin B_1_) and separated coculture of *C. cinerea* (separated in coculture in dialysis tubes) and *Gongronella* sp. w5 using SAHX medium (Sucrose DL-asparagine HX; in which sucrose (7.5 g/L) substituted for fructose of FAHX) were performed according to Hu et al. (2019) and Liu et al. (2022). In all experiments, the time 0 h of cocultivation refers to the start of coculture when homogenized *Gongronella* sp. w5 mycelium was added into the free medium of a 36-h-old culture of *C. cinerea* pregrown in a dialysis tube.

### Sequence and phylogenetic analysis of *Cc*AIF1 and *Cc*AIF2

The sequence similarity search of *Cc*AIF1 and *Cc*AIF2 was performed using NCBI BLASTP software (http://blast.ncbi.nlm.nih.gov/Blast.cgi). Multiple sequence alignment of AIF or AMID with homologous sequences from other species was performed using Clustal X 2.0 and Phylogeny fr3 (http://www.phylogeny.fr/index.cgi). The phylogenetic tree was constructed using MEGA 7 based on the neighbor-joining method (Kumar et al. 2016).

### Gene function assays in yeast

The coding region of *Cc*AIF1 was introduced into a *Hin*d III/*Bam*H I-digested yeast expression vector pYES2CT (presented by Professor Fan Yang in Dalian Polytechnic University) under the control of the yeast *GAL1* promoter. The resultant recombined vector and pYES2CT were used to transform the strain *S. cerevisiae* Y1HGold by the lithium acetate method to give a Y1H-*Cc*AIF1 strain and a Y1H-vector strain, respectively. The two transformants were then spotted on a synthetic drop-out SD-glucose plate or an SD-galactose plate and incubated at 30 °C for 3 days.

For DAPI (4,6-diamidino-2-phenylindole) staining of yeast nuclei, cells cultured for 36 h were collected, resuspended in 100% (v/v) methanol for brief fixation and permeabilization, and then stained for 15 min in the dark according to the manufacturer’s instructions (Beyotime Biotech, Shanghai, China). Cell images were taken using a laser confocal microscope (Olympus, Tokyo, Japan) at 364 nm excitation and 454 nm emission wavelengths.

For the cellular localization assay, a Y1H-GFP-*Cc*AIF1 strain was obtained by first fusing the coding region of *gfp* in frame to the 5′-end of gene *Ccaif1* (Elguindy and Nakamaru-Ogiso 2015; Ma et al. 2016), cloning the fragment into pYES2CT and transforming the construct into yeast as described above. Cells grown for 3 d on SD-galactose medium were incubated with 20 ng/mL Mitotracker Red CMXRos (Beyotime Biotech, Shanghai, China) for 15 min, washed twice with PBS (pH 7.4), and images were taken using a laser confocal microscope at 488 nm and 594 nm emission wavelengths, respectively (Akgul et al. 2000).

To verify that *Ccaif1* expression in yeast caused DNA fragmentation, TUNEL (TdT-mediated dUTP nick-end labeling) staining and the comet assay were used. The cells were grown on SD-glucose and SD-galactose medium for 3 days, respectively. For TUNEL staining, the collected cells were rinsed with PBS and fixed with 4% formaldehyde for 30 min. Then, the cells were resuspensed in PBS (pH 7.4) buffer containing 0.3% Triton X-100 and incubated at room temperature for 5 min. After washing with PBS twice, the cells were incubated with the TUNEL detection buffer (Beyotime Biotech, Shanghai, China) at 37 °C away from light for 60 min. The images were taken using a laser confocal microscope at 594 nm emission wavelengths. For the comet assay, normal melting point agarose was coated on the pretreated slides first. Then, the cell suspension was mixed with low melting point agarose at 37 °C and coated on the slides containing normal melting point agarose. The slides were treated with lysis solution (Beyotime Biotech, Shanghai, China) for 60 min at 4 °C, followed by gel electrophoresis. Subsequently, the slides were neutralized, stained with PI solution at room temperature, and observed under a fluorescence microscope at 594 nm emission wavelengths.

### Construction of *Ccaif1* silencing, *Ccaif1 *overexpression, and *gfp*-*Ccaif1 *overexpression *C. cinerea* strains

Total RNA was extracted from *C. cinerea* using the RNAiso Plus extraction reagent (TaKaRa, Dalian, China) according to the manufacturer’s protocol, followed by RNase-free DNase digestion (Promega, Beijing, China). One microgram of total RNA was used as the template for cDNA synthesis using a PrimeScript RT reagent kit (TaKaRa, Dalian, China). A *Ccaif1* antisense silencing fragment comprising the gene sequence of 930 to 1146 bp and the full-length cDNA of *Ccaif1* were amplified using primers listed in Table S1 and inserted behind the *A. bisporus gpdII* promoter into plasmid pYSK7 (Kilaru et al. 2006b) through homologous recombination in Y1HGold, respectively, as described by Liu et al. (2022). The *gfp*-*Ccaif1* overexpression vector was constructed by fusing *egfp* to the full-length cDNA of *Cc*AIF1 at the N-terminus to maintain its right localization (Elguindy and Nakamaru-Ogiso 2015; Ma et al. 2016). The resultant *Ccaif1* silencing plasmid pYSK-si*Ccaif1*, *Ccaif1* overexpression plasmid pYSK-ov*Ccaif1*, or *gfp*-*Ccaif1* overexpression plasmid pYSK-ov*gfp*-*Ccaif1* was cotransformed with the selection vector p*Cc*Ade8 into *C. cinerea* protoplasts according to Dörnte and Kües (2012). Transformants were selected on regeneration medium without adenine and further verified based on PCR amplification of the antisense *Ccaif1* or the full-length cDNA of *Ccaif1* using primers PF and PR (Table S1) (Dörnte and Kües 2012; Liu et al. 2022).

### RNA extraction and quantitative reverse transcription PCR (qRT-PCR) analysis

*C. cinerea* wild type and transformant mycelia from axenic cultures or separated cocultures at 0, 12, 24, 36, 48, 60, 72, 84, and 96 h incubation were employed for total RNA extraction. qRT-PCR was performed to analyze the transcript levels of *Ccaif1* and *lcc9* using a SYBR Green kit (TaKaRa, Dalian, China) on a Roche LightCycler 96 Real-Time PCR System (Roche, Basel, Switzerland). The relative expression levels were calculated using the 2^−∆∆CT^ method (Livak and Schmittgen 2001). The *β-actin* gene was chosen as the reference gene throughout the study (Liu et al. 2022). Gene expression analysis was performed for all clones on three parallel cultures each, with measurements in triplicate for each individual biological sample.

### Apoptotic-like PCD assays and localization detection of *Cc*AIF1 in *C. cinerea*

Apoptotic-like PCD was measured under dark conditions using an Annexin V-PI apoptosis detection kit (Keygen Biotech, Nan Jing, China) in *C. cinerea* wild type, *Ccaif1* silencing, and *Ccaif1* overexpressing cells. Briefly, the *C. cinerea* mycelia were first washed with PBS, then suspended in a 500-µL Annexin-V binding buffer to which 5 µL Annexin V-EGFP and 5 µL PI were added, and for 20 min incubated at room temperature. Finally, the fluorescence intensity of the samples was detected using a laser confocal microscope (Olympus, Tokyo, Japan) at 488 nm and 594 nm wavelengths.

Nuclear DNA fragmentation of the *C. cinerea* cells was assessed by DAPI staining. *C. cinerea* and *Gongronella* sp. w5 were inoculated on opposite sides of microscope slides with SAHX solid medium and grown for 4 days until the mycelia of two strains touched each other. The *C. cinerea* mycelia were fixed in 100% (v/v) methanol at room temperature for 5 min, washed with PBS (pH 7.4), stained with 2 µg/mL DAPI (Beyotime Biotech, Shanghai, China) for 3 min in the dark, and examined with a laser confocal microscope.

For the localization assay in *C. cinerea*, the GFP-*Cc*AIF1 overexpression *C. cinerea* transformant and *Gongronella* sp. w5 were cocultured on microscope slides with SAHX agar medium. The hyphae were stained with 20 ng/mL Mitotracker Red CMXRos for 15 min and photographed.

### Mycelial growth on agar plates, spore counting, and sensitivity to chemicals

*C. cinerea* wild type and *Ccaif1* silencing strains were inoculated with mycelial plugs (5 mm in diameter) onto solid SAHX (for coculture) or FAHX (for axenic culture) medium in the middle of the plates and incubated at 37 °C (Hu et al. 2019; Liu et al. 2022). In coculture, the initial distance between the inocula of *C. cinerea* and *Gongronella* sp. w5 on plates was about 5 cm. All *C. cinerea* clones were tested in axenic culture and in coculture on three parallel plates. Colonies on all agar plates were photographed daily and measured every 24 h to evaluate the mycelial growth rate of clones. The pictures were transformed into greyscale maps, and the colony areas were calculated by pixel scale using Matlab software (MathWorks Inc., MA).

The cocultures on agar plates were incubated in the dark at 37 °C for 7 days for abundant constitutive oidia production (Kües et al. 1998). The spores of the entire *C. cinerea* colonies were harvested from plates using sterile water by scraping the mycelium and were then counted using a haematocytometer.

To test the sensitivity of mycelia to chemicals, 100 mM H_2_O_2_ (Sangon Biotech, Shanghai, China) or 1 mM acetic acid (Sangon Biotech, Shanghai, China) was added to FAHX and SAHX agar plates for axenic cultures and cocultures, respectively. Mycelial growth rates were determined as described above.

All growth experiments were performed independently three times, always in triplicate plates per test case and run.

### Reactive oxygen species (ROS) and H_2_O_2_ assays

Intracellular ROS levels were measured using the fluorogenic probe DCFH-DA (2,7-dichlorodihydrofluorescein diacetate) (Beyotime Biotech, Shanghai, China). H_2_O_2_ levels were detected using H_2_O_2_ assay kits (Beyotime Biotech, Shanghai, China) as described in detail by Liu et al. (2022). *C. cinerea* wild type and mutant transformant from separated cocultures were harvested at 0, 12, 24, 36, 48, 60, 72, 84, and 96 h of incubation for ROS and H_2_O_2_ concentration assays. All experiments were performed independently three times and all samples were examined in triplicate.

### Laccase assay and native polyacrylamide gel electrophoresis (native-PAGE)

Aliquots of culture broth from separated cocultures were withdrawn every 12 h for laccase activity detection using 2,2′-azino-bis(3-ethylbenzothiazoline-6-sulfonate) (ABTS) (0.5 mM) as the substrate, following Bourbonnais and Paice (1990). Native-PAGE was performed on 12% polyacrylamide gels, as described by Pan et al. (2014). Gels were incubated at 25 °C in the citrate-phosphate buffer (pH 4.0) containing 1 mM ABTS for about 0.5 h and photographed using a digital camera.

The wild type and *Ccaif1* overexpression transformants were cultured in axenic culture in FAHX medium, and 1 mM H_2_O_2_ was added at 24 h of cultivation. Laccase activity in culture supernatants was determined with ABTS every 24 h and visualized on native-PAGE.

### LC-MS experiments

Protein bands with laccase activity werecollected from native-PAGE gels, and proteins in gels were trypsin-digested for LC-MS analysis (Applied Protein Technology, Shanghai, China). The MaxQuant software (v.1.5.3.17) was used for MS data analyses (https://www.maxquant.org/). Proteins were identified by searching the data against the annotated genome of *C. cinerea* downloaded in the GenBank database (Stajich et al. 2010).

### Statistical analyses

All experimental data were presented as mean ± standard deviation (SD). Statistical significance was evaluated by one-way ANOVA followed by Student’s *t*-test with GraphPad Prism 7.0 (GraphPad Software, Boston, MA, USA). *p* < 0.05 was considered statistically significant.

## Results

### Apoptotic-like PCD and increased transcription of a potential AIF are observed in *C. cinerea *during interspecific interaction with *Gongronella* sp. w5

Previous comparative transcriptomic analysis using *C. cinerea* Okayama 7 mycelia separated in dialysis tubes while cocultured with *Gongronella* sp. w5 for 18 h and 28 h, respectively, revealed expression of defense strategies in *C. cinerea* during fungal-fungal interactions (Liu et al. 2022). Intracellular ROS were upregulated and acted as signal molecules to stimulate defense responses by expressing various detoxification proteins. Simultaneously, two upregulated DEGs (differentially expressed genes), CC1G_08456 (2.17-fold at 28 h compared with 18 h) and CC1G_10894 (2.36-fold at 28 h compared with 18 h), were annotated to K22745 (apoptosis-inducing factor 2, AIF2), suggesting apoptotic-like PCD occurred in *C. cinerea* when interacting with *Gongronella* sp. w5. These two genes were named *Ccaif1* and *Ccaif2*, respectively.

**Fig. 1:**
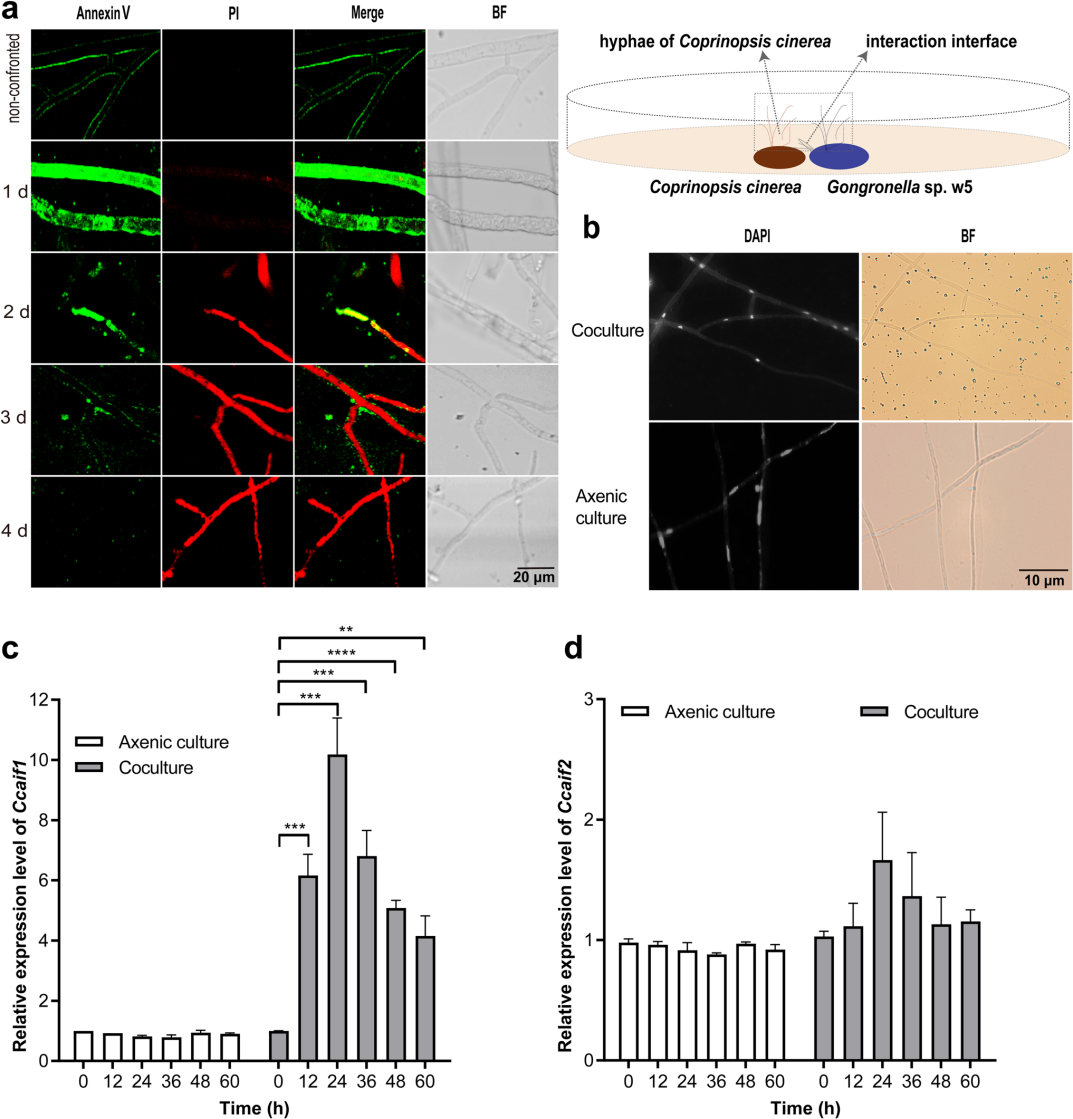
Apoptotic-like cell death is induced in *C. cinerea* (**a**, **b**) accompanied by upregulation of one identified AIF (**c**, **d**) in coculture of *C. cinerea* and *Gongronella* sp. w5. **a** *C. cinerea* mycelial growth fronts in the interspecific region with *Gongronella* sp. w5 on SAHX plates were costained with Annexin V–PI and analyzed under a fluorescence microscope. Annexin V is colored in green and PI is stained in red in the photos. Scale bars, 20 µm. **b** *C. cinerea* mycelia were stained with DAPI and showed increased nucleic DNA fragmentation during fungal-fungal interactions. Scale bars, 10 µm. **c** The AIF-annotated gene *Ccaif1* in *C. cinerea* was upregulated in transcriptional levels in liquid separated coculture of *C. cinerea* and *Gongronella* sp. w5. **d** The AIF-annotated gene *Ccaif2* in *C. cinerea* was not changed in transcriptional levels in liquid separated coculture of *C. cinerea* and *Gongronella* sp. w5. *C. cinerea* mycelia were collected, ground, extracted for RNA, and analyzed by qRT-PCR every 12 h. The transcriptional profiles of both annotated AIF genes, including *Ccaif1* and *Ccaif2*, were determined. *β*-actin was used as the control and the *Ccaif1* or *Ccaif2* transcriptional level of the wild-type strain at 0 h in axenic culture was set as the baseline. The data were analyzed using a Student *t*-test (significant statistical differences: **p < 0.01, ***p < 0.001, ****p < 0.0001). Data present means ± SD, *n* = 3. BF, Bright field

To verify this hypothesis, *C. cinerea* mycelia at extension fronts were firstly double-stained with the two markers Annexin V and PI that bind to externalized phosphatidylserine (PS) and nuclei, respectively. Scheme of the cell morphology assay on microscope slides of *C. cinerea* strains interacted with *Gongronella* sp. w5 was shown in Fig. 1a. The mycelia were strongly stained by Annexin V at 1–3 days of coculture (Fig. 1a), indicating exposure of PS on the outer leaflet of the plasma membrane and cells undergoing apoptotic-like PCD. Red staining observed at 2–4 days of coculture (Fig. 1a) indicated that PI entered the cells upon cell membrane disintegration and confirmed the onset of PCD. Furthermore, decreased staining of Annexin V and increased staining of PI occurred upon prolonged confrontation. Compared to cells with compact well-defined nuclei in the axenic culture, the nuclei of *C. cinerea* during fungal-fungal interactions harbored diffuse or fragmented DNA (Fig. 1b). Secondly, qRT-PCR was performed to confirm whether the two identified DEGs correlated with the induction of apoptotic-like PCD. As shown in Fig. 1c, the two DEGs had no change in expression in the axenic cultures of *C. cinerea* over the time, whereas in *C. cinerea* and *Gongronella* sp. w5 cocultures, *Ccaif1* enhanced in transcription at 12 h and peaked to 10.2-fold at 24 h, while by the end of cultivation, it was still being better expressed than in axenic cultures. In contrast, *Ccaif2* showed no significant change or only marginal variation (at 24 h) in expression throughout cultivation in axenic culture as in coculture. These results suggested that *Ccaif1* may play essential roles in apoptotic-like PCD induction in *C. cinerea*.

### Phylogeny and secondary structure analysis of *Cc*AIF1

**Fig. 2:**
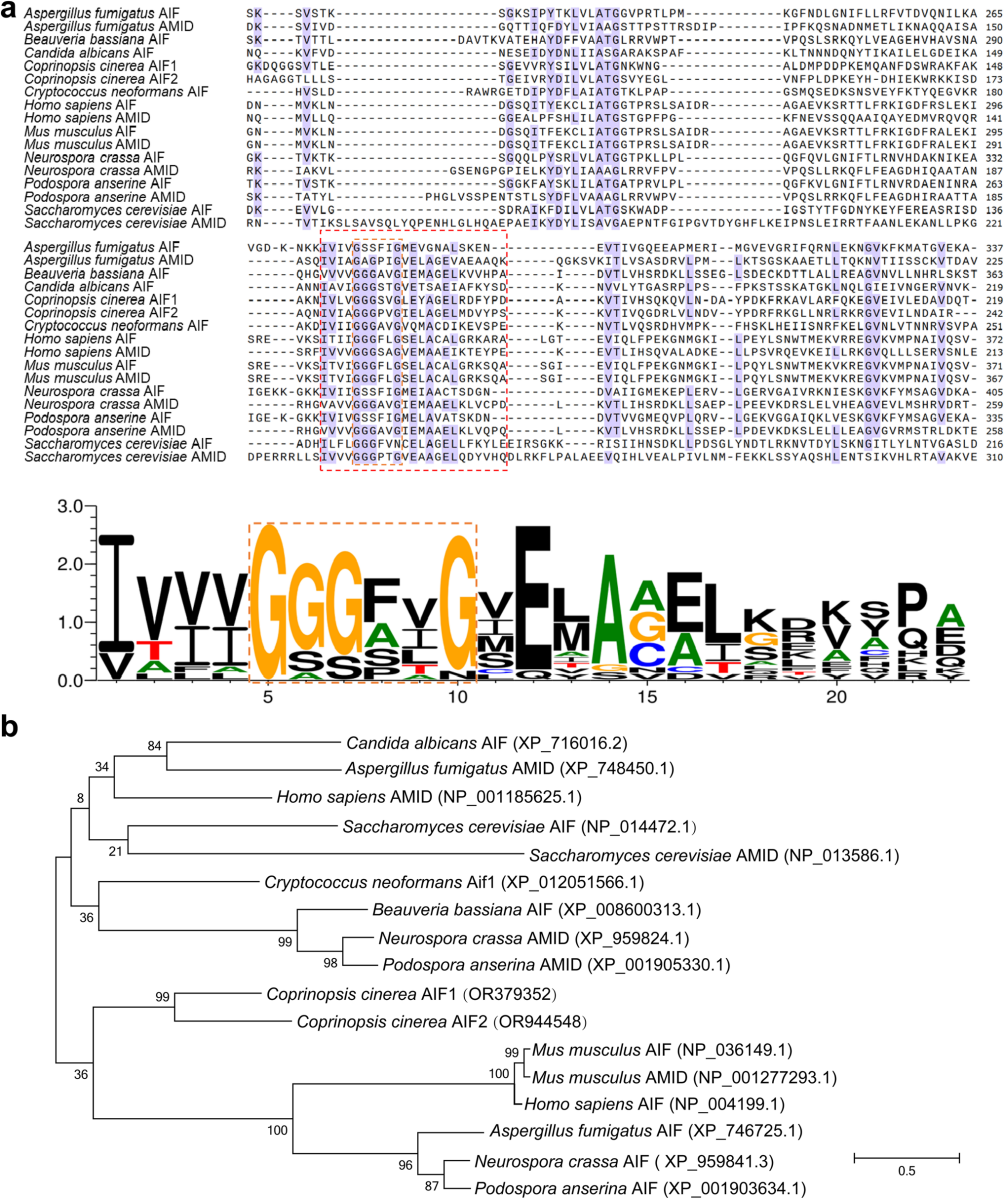
*Cc*AIF1 is an AIF homolog in *C. cinerea*. Amino acid alignment (**a**) and phylogenetic analysis (**b**) of *Cc*AIF1 and *Cc*AIF2 (accession nos. OR379352 and OR944548 in NCBI, respectively) with different AIF or AMID homologs from other species. Purple boxes in **a** indicate high amino acid identity (> 60% between all proteins); all accession numbers are provided in **b,** and the conserved Pyr_redox domain (Crooks et al. 2004) was marked in the dashed red box followed by drawing with WebLogo (the amino acids involving in NADH binding were marked in the dashed orange box). The phylogenetic tree in **b** was constructed using MEGA 7 (Kumar et al. 2016) based on the neighbor-joining method

*Ccaif1* is a gene with three introns localized on chromosome 12. Mature transcripts have an open reading frame of 1149 bp. The deduced mature protein consists of 382 amino acids (aa). *Cc*AIF1 (accession No. OR379352 in NCBI) has a conserved Pyr_redox domain and belongs to the pyridine nucleotide-disulfide oxidoreductase superfamily (Fig. 2a), whose members all contain nicotinamide adenine dinucleotide phosphate [NAD(P)] binding sites (Fig. 2a) embeded in sequences interacting with FAD (Li et al. 2022). BLASTP analysis in the NCBI database and the Uniprot database showed that *Cc*AIF1 shared sequence similarity with AIF and AMID homologs analyzed from other eukaryotic species, with the highest sequence similarity of 50% to *C*. *neoformans* Aif1. Furthermore, *Cc*AIF1 showed a sequence identity of 25.94% with human AMID and 20% with *C. albicans* AIF1 (Fig. 2a). Homology analysis using MEGA software confirmed that *Cc*AIF1 had a closer relationship with Aif1 identified from the unicellular basidiomycete *C*. *neoformans* than with the homologs from other species (Fig. 2b). Psort II (https://psort.hgc.jp/form2.html) analysis predicted for *Cc*AIF1 an expected mitochondrial location (52.2%), with a potential presequence processing site IRT|TV at aa 64 and a putative NLS (nuclear localization signal) at aa position 192-198 (PDKFRKA).

### *Cc*AIF1 localizes in mitochondria and its overexpression in yeast induces apoptosis

**Fig. 3:**
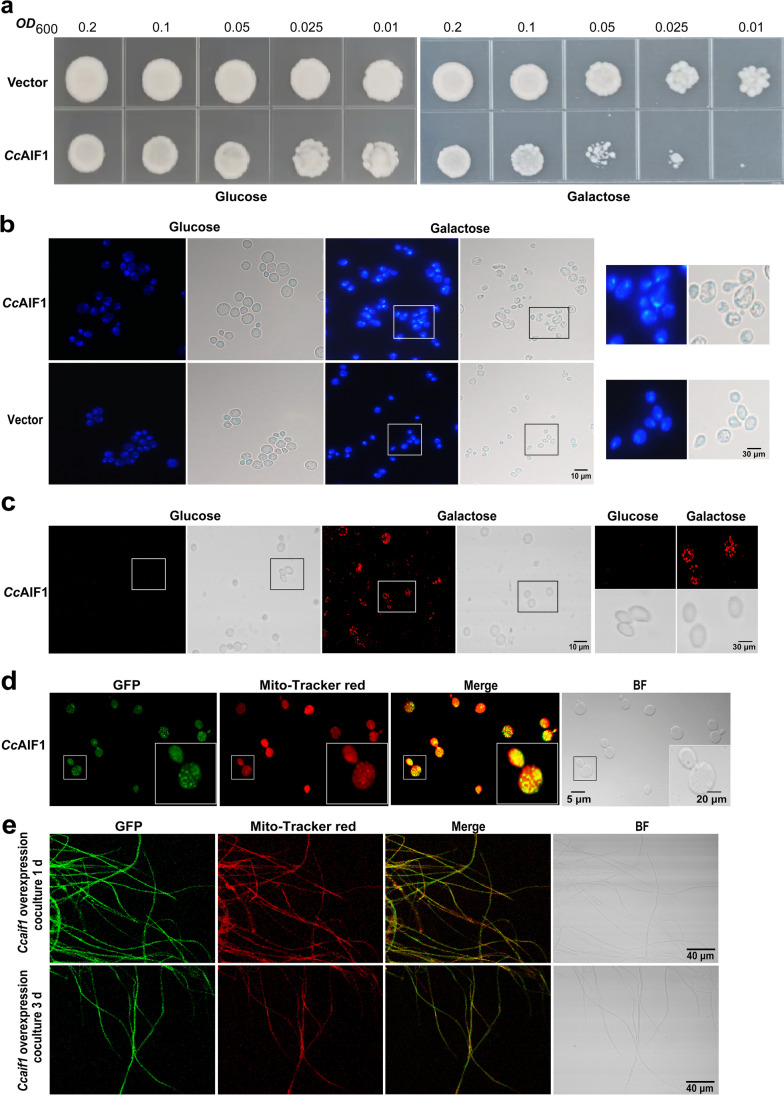
*Cc*AIF1 localizes in mitochondria and its overexpression induces apoptosis in the yeast *S. cerevisiae*. **a**–**c** *Ccaif1*-overexpression yeast cells display a growth defect (**a**), chromatin condensation (**b**), and DNA fragmentation (**c**). Yeast cells were spotted in serial dilutions onto corresponding positions of an SD-glucose plate or on an SD-galactose plate for *GAL* promoter induction and incubated for 3 days at 30 °C. Then, the cells were collected and stained by DAPI or examined by the TUNEL assay. *Cc*AIF1, *Ccaif1* overexpression yeast strain. Vector, yeast strain transformed with the empty vector. Scale bars, 10 and 30 µm, respectively. **d**, **e** *Cc*AIF1 localizes in mitochondria. Y1H-GFP-*Cc*AIF1 cells (**d**) and GFP-*Cc*AIF1 overexpression transformant of *C. cinerea* confronted with *Gongronella* sp. w5 (**e**) were stained with the mitochondria marker Mitotracker Red CMXRos and then analyzed under a fluorescence microscope. Scale bars, 5, 20, and 40 µm, respectively

*Cc*AIF1 was heterologously expressed in *S. cerevisiae* Y1H behind the yeast *GAL1* promoter to further explore its physiological function. The resultant yeast Y1H-*Cc*AIF1 strain grew well on a glucose-containing SD medium. In contrast, once it was cultured on a galactose medium to trigger *Cc*AIF1 expression, the cell viability decreased (Fig. 3a). DAPI staining showed that the number of cells showing chromatin condensation increased from 10 to 73% after adding galactose for 48 h based on counts of 500 cells each (Fig. 3b). The fragmentation of DNA was further demonstrated by both TUNEL staining and comet experiments (Fig. 3c; Fig. S1). In addition, galactose-cultured Y1H-*Cc*AIF1 cells were often larger and their cell surface wrinkled, similarly as described before for cells overexpressing the native yeast AMID (Li et al. 2006). In comparison, nuclei of cells transformed with plasmid pYES2CT were regular and homogenous in shape, even when grown on galactose medium (Fig. 3b). These results together suggested that *Ccaif1* overexpression caused apoptosis in yeast cells. Further localization assay using the Y1H-GFP-*Cc*AIF1 strain and the GFP-*Cc*AIF1 overexpression *C. cinerea* transformant revealed that GFP-*Cc*AIF1 was mainly located in mitochondria, in line with the PSORT II prediction (Fig. 3d and e). However, upon prolonged confrontation with *Gongronella* sp. w5, GFP-*Cc*AIF1 only partially colocalized with the mitochondria marker, indicating its translocation from the IMS to the cytoplasm in *C. cinerea* during apoptotic-like PCD.

### *Ccaif1* silencing inhibits but overexpression promotes the apoptotic-like PCD in *C. cinerea* during coculture

*C. cinerea* protoplasts were transformed with *Ccaif1* antisense silencing plasmid or *Ccaif1* overexpression plasmid to investigate whether *Cc*AIF1 was involved in apoptotic-like cell death in the native host during fungal interspecific interaction. Twenty silencing transformants and four overexpression transformants were obtained in cotransformation using p*Cc*Ade8 and adenine as the selection marker, followed by PCR amplification of the inserted *Ccaif1* antisense or *Ccaif1* cDNA fragment, respectively, as proof. Based on the qRT-PCR results, the transcriptional level of *Ccaif1* for all the 20 potential silencing transformants was 50–85% silenced in axenic culture compared to that observed in the wild-type strain (Fig. S2a). Furthermore, three overexpression strains exhibited three- to fivefold increases in *Ccaif1* transcription level (Fig. S2b).

**Fig. 4:**
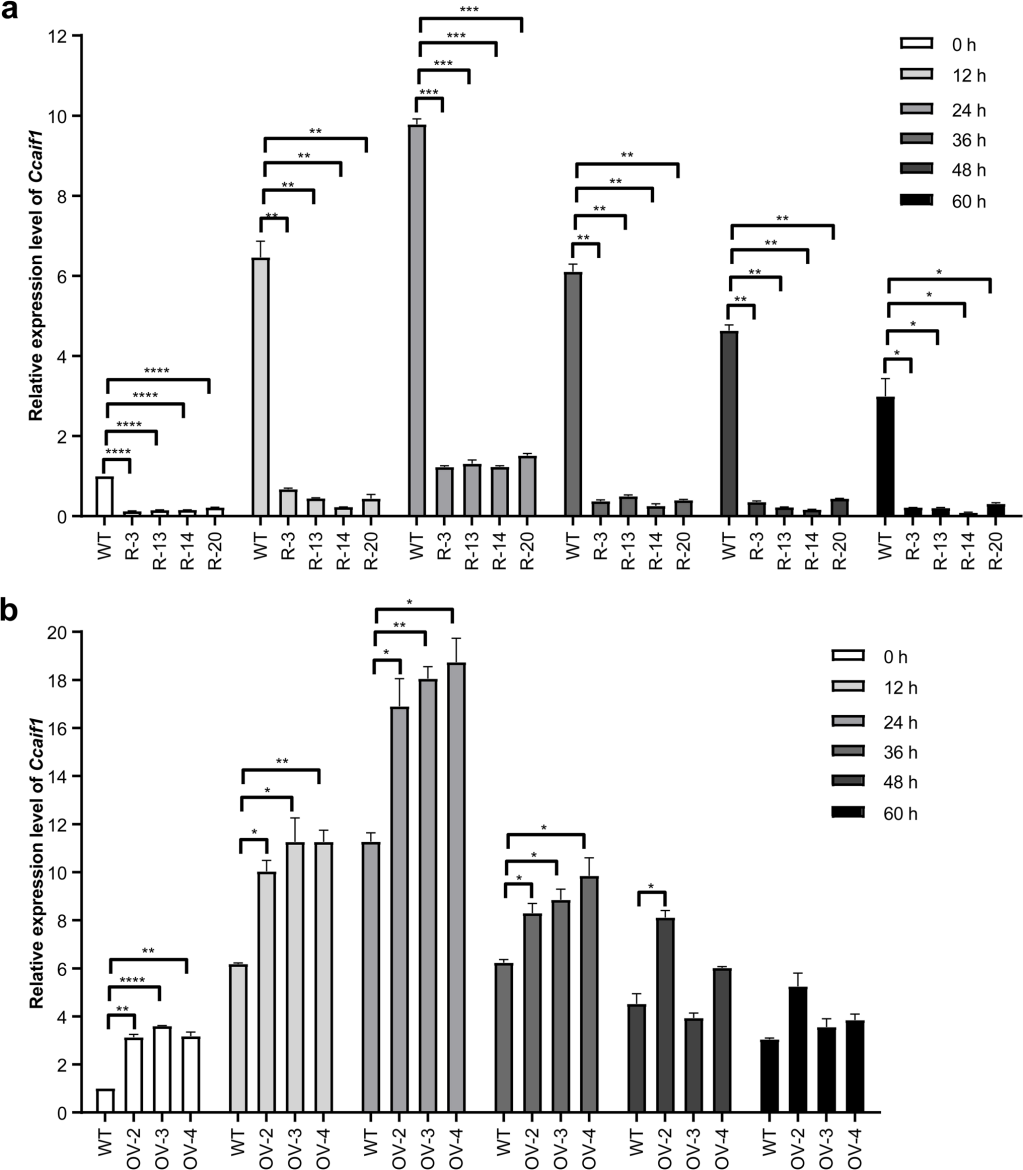
*Ccaif1* is downregulated in the four selected *Ccaif1* silencing transformants R-3, R-13, R-14, and R-20 (**a**) and upregulated in the three *Ccaif1* overexpression transformants OV-2, OV-3, and OV-3 (**b**) of *C. cinerea*, during *C. cinerea*-*Gongronella* sp. w5 interspecific interaction in separated coculture. *C. cinerea* mycelia were collected every 12 h and *Ccaif1* transcripts detected by qRT-PCR analysis. *Β*-*actin* was used as the control and the *Ccaif1* transcriptional level of the wild-type strain at 0 h was set as the baseline. The data were analyzed using a student’s *t*-test (significant statistical differences: *p < 0.05, **p < 0.01, ***p < 0.001, ****p < 0.0001). Data present means ± SD, *n* = 3. WT, untransformed *C. cinerea* wild type

Four silencing strains named R-3, R-13, R-14, and R-20 exhibiting more than 75% interference efficiency and three overexpression strains named OV-2, OV-3, and OV-4 were chosen in the following coculture experiments. Similar to the axenic culture, transcriptional levels of *Ccaif1* in the four silencing strains decreased by about 75–85% compared to the wild-type strain at 0 h in separated coculture with *Gongronella* sp. w5 (Fig. 4a). During 0–60 h of coculture, *Ccaif1* transcripts were all significantly less upregulated in these silencing strains than in the wild-type strain (*p* < 0.01, *p* < 0.001, or *p* < 0.0001) (Fig. 4a). By comparison, the transcriptional levels of *Ccaif1* increased dramatically from 0 to 36 h in the three overexpression strains compared to that in the wild-type strain (Fig. 4b).

**Fig. 5:**
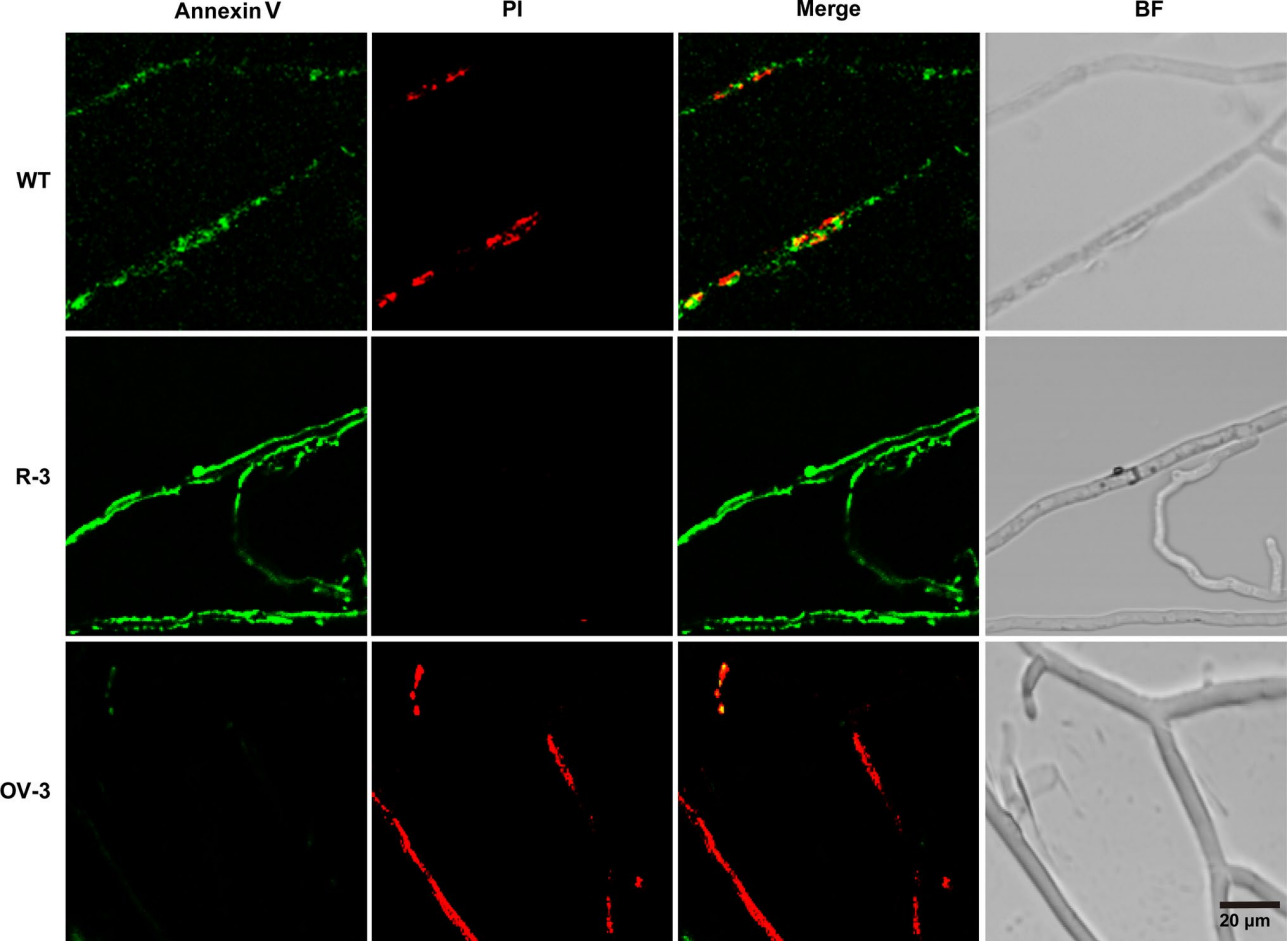
*Cc*AIF1 regulates apoptosis in *C. cinerea* under interspecific interaction with *Gongronella* sp. w5. The *C. cinerea* wild-type strain (WT), *Ccaif1* silencing transformant R-3, and *Ccaif1* overexpression transformant OV-3 were cocultured with *Gongronella* sp. w5 on SAHX medium for 2 days. Then, the *C. cinerea* mycelia at the confrontation side were stained with Annexin V–PI and analyzed under a fluorescence microscope. Scale bars, 20 µm. BF, bright field

Annexin V–PI staining was performed for the wild type, *Ccaif1* silencing, and *Ccaif1* overexpression *C. cinerea* mycelia at the confrontation side when interacting with *Gongronella* sp. w5 for 2 days on SAHX agar plates. The phenotypes of R-3 and OV-3 were randomly selected and presented in Fig. 5. Compared with the wild-type strain, *Ccaif1* silencing *C. cinerea* mycelia showed a slightly stronger intensity of fluorescence based on Annexin V staining but presented a substantially decreased fluorescence intensities of PI staining. In contrast, *Ccaif1* overexpression *C. cinerea* exhibited dramatically weaker fluorescence intensities of Annexin V staining than the other two types of mycelia. However, compared with the wild-type strain, the fluorescence intensities of PI staining were increased by *Ccaif1* overexpression. The mycelia from the non-confronted side of these strains were also stained and observed. No obvious difference occurred in either Annexin V or PI staining, which coincided with the result that *Ccaif1* was only highly triggered to express in coculture rather than in axenic culture (Fig. 1c). These results in consequence suggest that *Ccaif1* silencing inhibits, but overexpression promotes apoptotic-like PCD in *C. cinerea* during interactions with *Gongronella* sp. w5.

### *Cc*AIF1 is necessary for *C. cinerea* to confront oxidative stress in fungal-fungal interaction

**Fig. 6:**
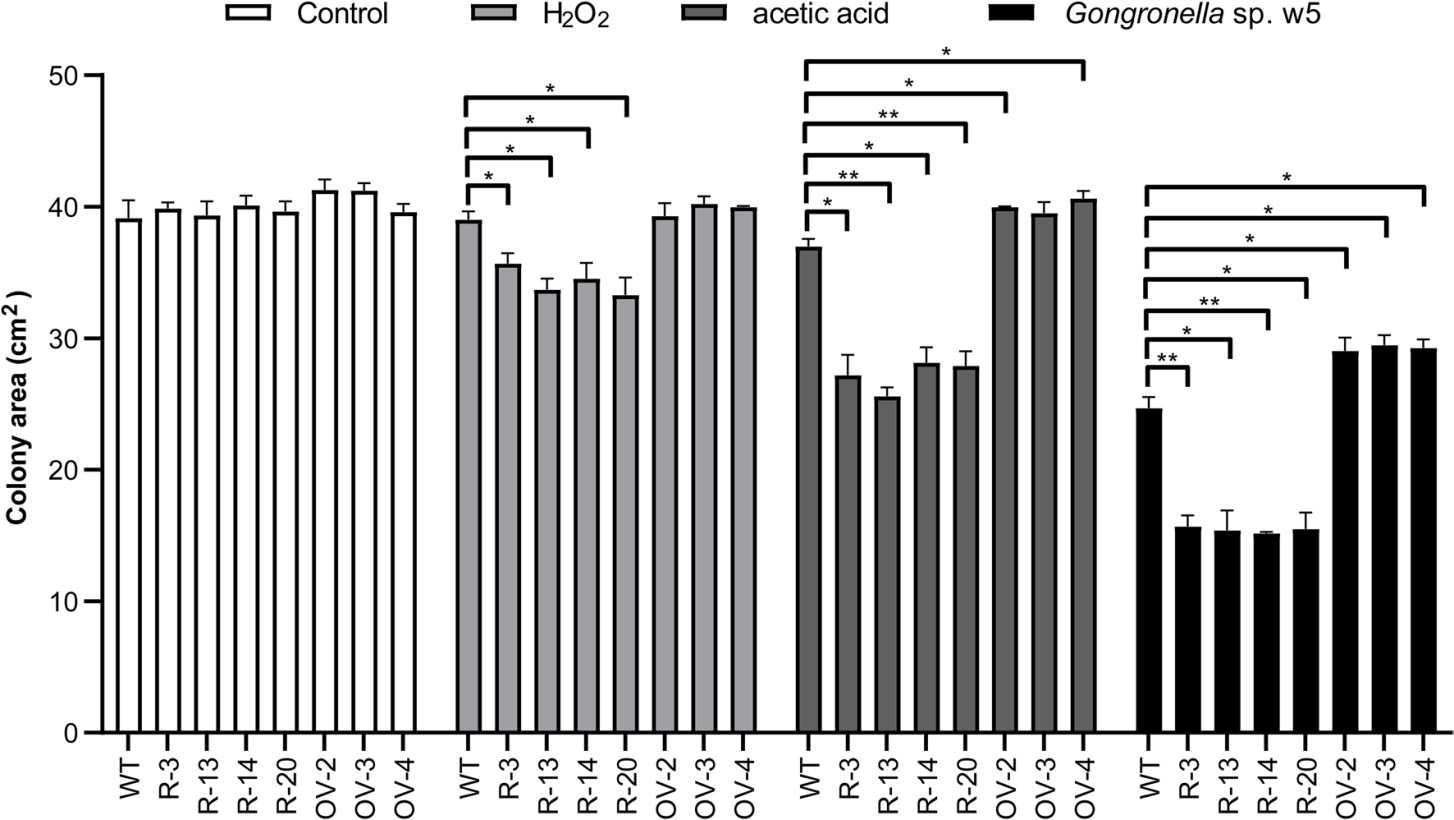
*Cc*AIF1 is involved in cell growth under oxidative stress. The wild-type (WT), *Ccaif1* silencing (R-3, R-13, R-14, R-20), and *Ccaif1* overexpression (OV-2, OV-3, OV-4) transformants of *C. cinerea* were cultured on FAHX agar plates adding 100 mM H_2_O_2_ or 1 mM acetic acid, or on SAHX agar plates coculturing with *Gongronella* sp. w5. The colony areas were quantified and analyzed using a student’s *t*-test (significant statistical differences: *p < 0.05, **p < 0.01). Data present means ± SD, *n* = 3

To examine the effect of *Cc*AIF1 on the oxidative stress confrontation in *C. cinerea* during it faced off with *Gongronella* sp. w5, the growth rate of the *C. cinerea* transformants was first measured in axenic cultures to elucidate their sensitivity to oxidative stress. All strains showed no significant difference in mycelial growth when cultured on FAHX agar plates without chemical addition. When exposed to 100 mM H_2_O_2_ or 1 mM acetic acid, *Ccaif1* silencing transformants showed higher sensitivity than the wild-type strain, and their growth expansion rates were significantly reduced. In contrast, the growth rates of *Ccaif1* overexpression transformants were faster than the other two types of strains (Fig. 6; Fig. S3). For example, after being exposed to 1 mM acetic acid for 5 days, the colony area of the wild-type strain was 36.99 ± 0.56 cm^2^, while in strains R-3 and OV-3, the colony areas were significantly different with 27.20 ± 1.55 cm^2^ (*p* < 0.05) and 39.50 ± 0.86 cm^2^ (*p* < 0.05), respectively.

Next, cocultures were performed to analyze the growth rate of wild type, *Ccaif1*-silencing, and *Ccaif1*-overexpression *C. cinerea* clones after antagonistic interaction with *Gongronella* sp. w5. The growth rate of *C. cinerea* was restricted after cocultivation on SAHX agar plates. *Ccaif1* silencing further slowed the growth rates of *C. cinerea*, but *Ccaif1* overexpression reversed the sensitivity of *C. cinerea* to *Gongronella* sp. w5. For instance, the colony area decreased from 24.70 ± 0.84 cm^2^ in the wild-type strain to 15.69 ± 0.85 cm^2^ (*p* < 0.01) in the transformant R-3 but increased to 29.48 ± 0.77 cm^2^ (*p* < 0.05) in the transformant OV-3 (Fig. 6). Moreover, the absolute number of oidia of the *C. cinerea* wild-type strain was much higher than that of the *Ccaif1* silencing transformants but lower than that of the *Ccaif1* overexpression transformants after interacting with *Gongronella* sp. w5 for 7 days (Table S2). When calculating relative numbers (sum of oidia/colony area), differences were encountered but less pronounced, suggesting both effects by expression levels of *Cc*AIF1 and by altered growth speed on oidia production.

**Fig. 7:**
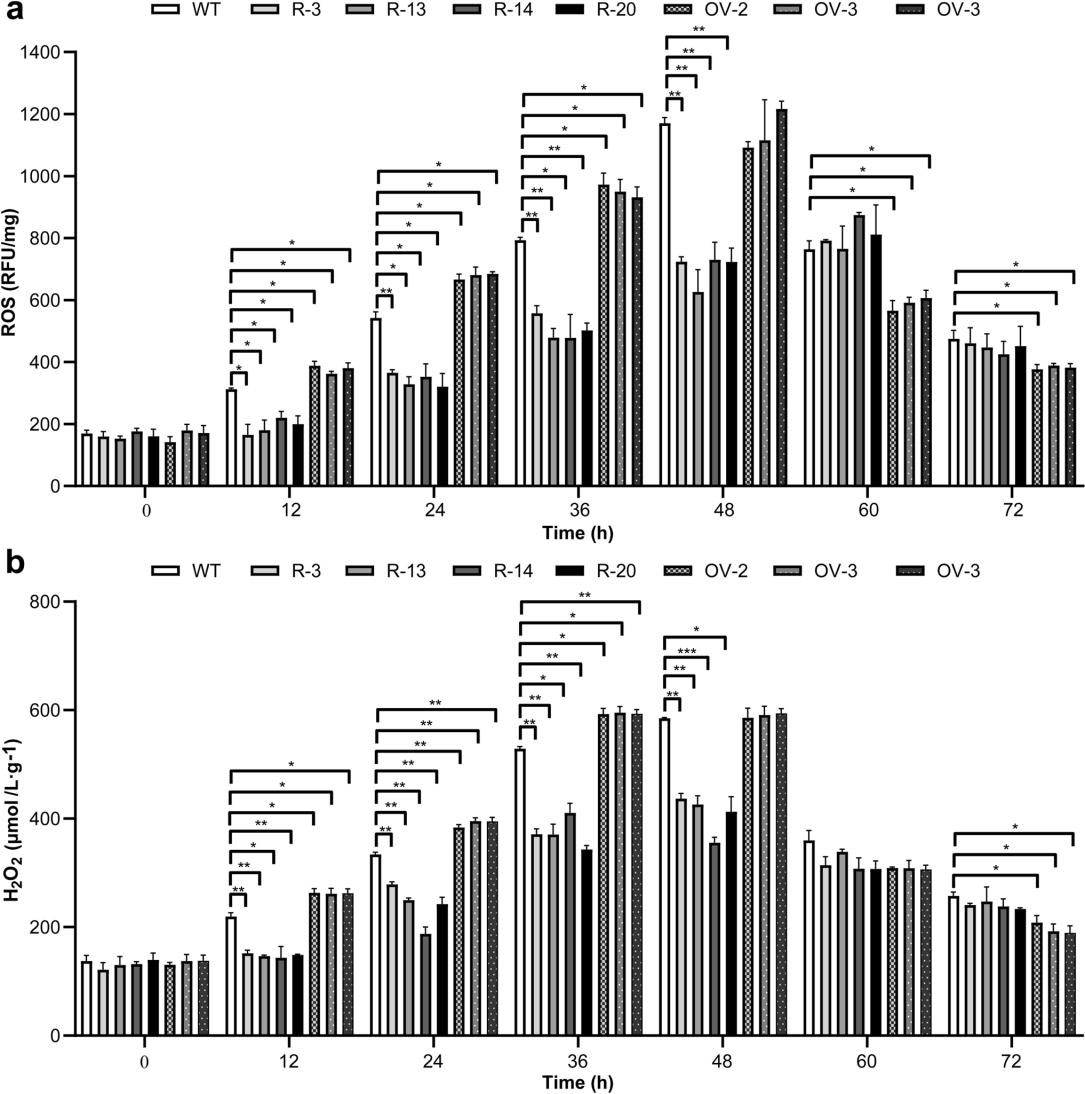
*Cc*AIF1 regulates the intracellular ROS levels and H_2_O_2_ contents in *C. cinerea* during interspecific interaction with *Gongronella* sp. w5. ROS levels (**a**) and H_2_O_2_ contents (**b**) in *Ccaif1* silencing (R-3, R-13, R-14, R-20) and *Ccaif1* overexpression (OV-2, OV-3, OV-4) transformants of *C. cinerea* were compared to the untransformed wild-type (WT) strain every 12 h grown in separated cocultures with *Gongronella* sp. w5. The data were analyzed using a student’s *t*-test (significant statistical differences: *p < 0.05, **p < 0.01, ***p < 0.001). Data present means ± SD, *n* = 3

The intracellular ROS and H_2_O_2_ concentrations of the wild-type, *Ccaif1*-silencing, and *Ccaif1*-overexpression *C. cinerea* clones were tested and compared during their separated coculture with *Gongronella* sp. w5 to examine the effect of *Cc*AIF1 on the oxidative stress confrontation in *C. cinerea*. The four selected silencing transformants showed 30–50% decreased ROS concentrations and 17–45% decreased H_2_O_2_ concentrations compared to the parental wild-type strain during the first 12–48 h of cocultivation, whereas no significant differences were observed among the cultures at 60 and 72 h of incubation (Fig. 7). In the three overexpression transdformants, 15–25% increased ROS levels and 12–20% increased H_2_O_2_ concentrations were observed at 12–36 h of cultivation. However, after 60 h and 72 h of coculture, their ROS levels were lower than that in the wild-type strain (Fig. 7).

### *Cc*AIF1 regulates laccase Lcc9 activation in *C. cinerea* during fungal-fungal interaction

**Fig. 8:**
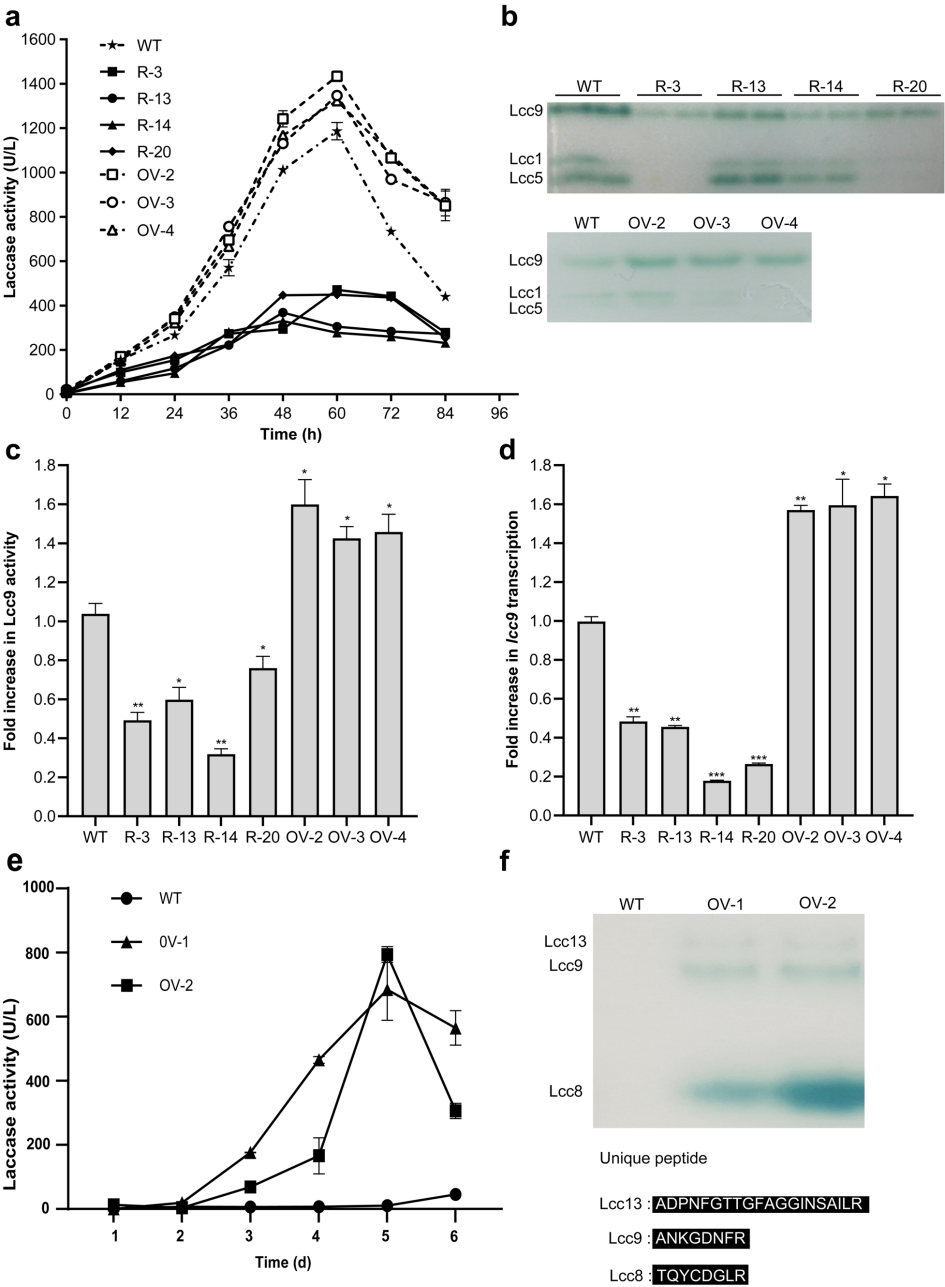
*Cc*AIF1 overexpression not only increases Lcc9 expression in coculture with *Gongronella* sp. w5 but also upregulates Lcc8 and Lcc13 expression in axenic culture when exposed to H_2_O_2_. **a** The laccase activity of the *C. cinerea* wild-type (WT) strain, *Ccaif1* silencing transformants (R-3, R-13, R-14, R-2), and *Ccaif1* overexpression transformants (OV-2, OV-3, OV-4) were tested over the cultivation time in separated coculture with *Gongronella* sp. w5. **b** Native-PAGE of laccase activities in *C. cinerea* clones at 60 h incubation of separated coculture with *Gongronella* sp. w5. Equal amounts of culture supernatants (10 µL) were loaded on the gels and activity-stained after gel-electrophoresis. **c** Quantification of Lcc9 activities deduced from gels as shown in panel **b**. **d** *lcc9* transcripts were measured by qRT-PCR analysis in *C. cinerea* clones at 36 h incubation in separated coculture with *Gongronella* sp. w5. The *lcc9* transcription level of the wild-type (WT) *C. cinerea* strain at 36 h incubation in axenic culture was set as the baseline. **e** The laccase activity of the wild-type strain, and *Ccaif1* overexpression transformants OV-1 and OV-2 were measured in axenic culture with 1 mM H_2_O_2_ added. **f** Native-PAGE of laccase activities detected at 60 h incubation from panel **e**. The bands in the gels with laccase activity were cut and analyzed for MS to identify the individual laccase enzymes and identified lacceses are marked. The data in **c** and **d** were analyzed using a Student *t*-test (significant statistical differences: *p < 0.05, **p < 0.01, ***p < 0.001). Data present means ± SD, *n* = 3

Previously, we demonstrated that ROS acted as signal molecules for the enhanced laccase Lcc9 production in *C. cinerea* when interacting with *Gongronella* sp. w5 (Liu et al. 2022). Therefore, the changes in ROS concentration in the abovementioned *C. cinerea* transformants may be related to Lcc9 expression. Therefore, in the following, laccase activities and transcripts were compared in transformants and the wild type during separated cocultivation with *Gongronella* sp. w5. As shown in Fig. 8a, compared with the wild-type strain, all four silencing transformants exhibited 60–75% lower laccase activities over the whole coculture time, whereas the three overexpression transformants presented higher laccase activities. Native-PAGE of coculture supernatant samples at 60 h showed Lcc9 expression was significantly affected by *Ccaif1* silencing or overexpression (*p* < 0.05 or *p* < 0.01) (Fig. 8b and c). In addition, Lcc1 and Lcc5 activities were also decreased in three of the *Ccaif1* silencing transformants of the four selected *Ccaif1* silenced transformants (Fig. 8b). The *lcc9* transcriptional level showed 50–80% downregulation in *Ccaif1* silencing transformants and 55–75% upregulation in *Ccaif1* overexpression transformants (Fig. 8d).

To further illustrate whether *Cc*AIF1-mediated ROS signals could promote Lcc9 expression also in axenic cultures, the laccase activities were compared between the wild-type strain and two randomly chosen *Ccaif1* overexpressing strains in response to 1 mM H_2_O_2_. The total laccase activity of the wild-type strain did not change when exposed to H_2_O_2_, whereas it was upregulated hundreds of times in the transformants OV-1 and OV-2. Specifically, the maximum activity was 684 U/L and 794 U/L in OV-1 and OV-2 cultures at 5 days of incubation, respectively (Fig. 8e). Furthermore, native-PAGE and LC-MS analysis inferred that Lcc9 was more strongly expressed in the two *Ccaif1* overexpressing transformants in axenic culture than the wild-type strain. Unexpectedly, however, two isozymes were also expressed and identified through LC-MS analysis as Lcc8 and Lcc13 (Fig. 8f). Among them, Lcc8 contributed most of the laccase activity in the fermentation supernatant, more than Lcc9. Therefore, *Cc*AIF1 transmitted the H_2_O_2_ signals and regulates some laccase isozyme expression, including Lcc9 in *C. cinerea*. As unraveled by this work, *Cc*AIF1 overexpression combined with H_2_O_2_ stimulation is therefore a new and very effective strategy for high yield laccase production in cultures.

## Discussion

Several criteria have been reported for the consensus definition of apoptotic-like PCD in fungi (Shlezinger et al. 2012; Hardwick 2018; Herrmann and Riemer 2021), such as the PS externalization on the outer leaflet of the plasma membrane, chromatin condensation, DNA fragmentation, and pro-apoptotic proteins releasing from the IMS (Carmona-Gutierrez et al. 2018). Compared with the unicellular yeast, multicellular fungi typically form a network of interconnected cells sharing a common cytoplasm and organelles (Daskalov et al. 2017). Several studies have focused on apoptotic-like PCD in pathogenic multicellular fungi (Cheng et al. 2003; Dinamarco et al. 2011; Banoth et al. 2020; Chen et al. 2021), whereas only a few nonpathogenic multicellular fungal species, such as *C. cinerea* and *Pleutotus* sp., were reported to show typical chromatin condensation and DNA fragmentation after entry to meiotic metaphase (Lu et al. 2003) and exhibit nuclear condensation, ROS accumulation, and DNA fragmentation when exposed to heat stress (Song et al. 2014). These studies suggest that apoptotic-like PCD might exist in all multicellular fungi. However, the roles of apoptotic-like PCD in many biological processes and regulation mechanisms remain underexplored.

In this work, we identified *Cc*AIF1 as an apoptosis-inducing factor acting in apoptotic-like PCD and stress-regulated gene expression in *C. cinerea*. CcAIF1 has the structural hallmarks of AIFs and AMIDs known from other apoptotic systems, like a conserved Pyr_redox domain, and it is predicted by PSORT II to localize in mitochondria (Fig. 2). However, such predictions of localization for a potential AIF need to be backed up experimentally, as they can vary widely between different AIFs and AMIDs. *S. cerevisiae* Aif1p (378 aa), for example, has a potential proteolytic VRL|TV cleavage motif at aa 61. It is located in mitochondria and translocates to the nucleus in response to apoptotic stimuli (Wissing et al. 2004), although its localization is predicted by PSORT II to be endoplasmic reticulum (ER; 44.4%). In contrast, human AMID (373 aa) attaching to the outside of mitochondria (Wu et al. 2002) is predicted to lack a proteolytic cleavage site and to be cytoplasmic (60.9%). All three proteins have no apparent extra typical N-terminal bipartite MLS with membrane tether unlike the larger human Aif1 (613 aa; predicted mitochondrial location 39.1%; proteolytic cleavage motif TRQ|MA at aa 62; NLS PEQKQKK at aa 106-112) that can enter the IMS (Susin et al. 1999; Wu et al. 2002; Sevrioukova 2011) but has in addition been detected in the ER for transport into mitochondria (Chiang et al. 2012). Heterologous expression in yeast and overexpression in *C. cinerea* both indicated *Cc*AIF1 to localize to mitochondria (Fig. 3d and e). Upon expression, the yeast underwent apoptotic-like PCD (Fig. 3a) and overexpression and silencing of *Ccaif1* in the native host provided further evidence that *Cc*AIF1 acts in apoptotic-like PCD also in *C. cinerea* (Fig. 5).

PI is often costained with Annexin V to yield Annexin V/PI double-stained cells, marking the phenotypical shift from early stages in apoptotic-like PCD to secondary necrosis by cellular entry of PI faciliated by cellular membrane disintegration, which is classified as late apoptosis (Büttner et al. 2007; Rogers et al. 2017; Carmona-Gutierrez et al. 2018). In our study, when *C. cinerea* interacted with *Gongronella* sp. w5, the mycelia at extension fronts were strongly stained by Annexin V from 1 day of cultivation (Fig. 1a), along with the increased staining of PI and nucleic DNA fragmentation seen shortly after (Fig. 1a and b). Thus, apoptotic-like PCD occurs in *C. cinerea* during fungal antagonistic interactions. *Cc*AIF1 transcripts increased in *C. cinerea* wild-type strain throughout its coculture with *Gongronella* sp. w5 (Fig. 1c). At the same time, *Ccaif1* silencing inhibited and *Ccaif1* overexpression promoted the apoptotic-like PCD process (Fig. 5). The results from phylogeny analysis (Fig. 2b) and heterologous expression of *Cc*AIF1 in *S. cerevisiae* (Fig. 3) demonstrated furthermore that *Cc*AIF1 is an AIF homolog that drives apoptotic-like PCD. The induced apoptotic-like PCD is necessary for *C. cinerea* to antagonize *Gongronella* sp. w5 perhaps due to the removal of damaged cells, as silencing *Ccaif1* in transformants slowed down the growth rate of *C. cinerea*, while overexpression of *Ccaif1* reversed this phenotype (Fig. 6). Therefore, for the first time, we identified an AIF in multicellular basidiomycetes and demonstrated its important function involving in apoptotic-like PCD during multicellular fungal antagonistic interactions. However, the function of *Cc*AIF2, which harbored 60% sequence similarity with *Cc*AIF1, might not be related to fungal antagonism according to its unchanged transcriptional levels during coculture (Fig. 1d). As PCD was observed also in mycelial aging and fruting body development (Lu and Sakaguchi 1991; Shlezinger et al. 2012), these AIF paralogs might work in different physiological processes and act synergistically to maintain cellular homeostasis.

AIF or AMID members are not just apoptosis-inducing factors. They are characterized by an oxidoreductase domain involved in mitochondria metabolism, redox control, and stress confrontation (Urbano et al. 2005; Joza et al. 2009; Elguindy and Nakamaru-Ogiso 2015; Herrmann and Riemer 2021). Based on the secondary structure analysis, *Cc*AIF1 had a conserved Pyr_redox domain (Fig. 2a). *Ccaif1* silencing led to higher sensitivity to oxidative stress caused by chemicals or the presence of *Gongronella* sp. w5, whereas its overexpression resulted in stronger resistance of transformants compared to the wild-type strain of *C. cinerea* (Fig. 6). These results are consistent with observations on *A. nidulans aifA* (Savoldi et al. 2008), as well as the AMID homolog of *N. crassa*, the deletion of which resulted in reduced resistance against chemicals or H_2_O_2_ (Castro et al. 2008). In contrast, PaAIF2 and PaAMID2 are reported to be negatively related to oxidative stress tolerance in *P. anserina* (Brust et al. 2010). These discrepancies might be partially associated with the alternate localizations of AIF or AMID in cells (Brust et al. 2010). Similar to the mitochondria-localized *N. crassa* AIF (Castro et al. 2008) and *C. albicans* AIF1 (Ma et al. 2016), *Cc*AIF1 in this study was mitochondrial and positively regulated oxidative stress confrontation during coculture (Figs. 3 and 6).

Over 90% of cellular ROS are produced by mitochondria via the escape of electrons from the mitochondria electron transport system (D'Autréaux and Toledano 2007; Montibus et al. 2015). *Ccaif1* overexpression resulted in higher cellular ROS and H_2_O_2_ levels than in the wild-type *C. cinerea* strain at 12–36 h of cultivation, corresponding with upregulated Lcc9 expression and activities in both coculture and axenic culture (Figs. 7 and 8). Thus, *Cc*AIF1 might affect the mitochondria respiratory complex and act as a ROS homeostasis controller. The results also reinforced our previous conclusion that ROS contributed to *lcc9* activation (Pan et al. 2014; Liu et al. 2022). Moreover, Lcc9 was demonstrated to be used as a powerful defense strategy to eliminate oxidative stress during fungal interactions (Liu et al. 2022). It was assumed that the induced laccase was responsible for the decreased ROS levels after 60 h of coculture in *C. cinerea* (Fig. 7). Upregulated PCD and secretion of Lcc9 together facilitated stress confrontation and enhanced cell growth of *C. cinerea* during interaction with *Gongronella* sp. w5.

Interestingly, in this study, it was observed for the first time that another two previously silent isozymes of the 17 distinct *C. cinerea* laccases (Kilaru et al. 2006a; Rühl et al. 2013; Pan et al. 2014), Lcc8 and Lcc13, were expressed from their native genes in axenic culture of *Ccaif1* overexpression strains treated with H_2_O_2_, with Lcc8 becoming the major isozyme (Fig. 8). Lcc8 has two predicted isoforms, including one of normal laccase length (567 aa) but without a predicted signal peptide and a “long” one (728 aa) with a signal sequence of 23 aa length at the N-terminus of an unusual 161 aa-long N-terminal protein extension (Schulze et al. 2019). Though Lcc8 is grouped into the same laccase subfamily as Lcc9, neither of the isoforms has been detected in *C. cinerea* wild-type cultures, probably due to the lack of splicing of a first postulated intron required to give the normal length Lcc8 version with an in-frame ATG start codon (Kilaru et al. 2006a). Adding the *C. cinerea lcc1* sequences for a functional signal peptide to the shorter basic *lcc8* coding sequence proved secreted expression under control of the *Agaricus bisporus gpdII* promoter of functional Lcc8 laccase in transformed *C. cinerea* (Schulze et al. 2019). Lcc8 in this study migrated in native gels below laccase Lcc9, similar to Lcc1 and Lcc5 (Fig. 8). Whether this indicates it might be the shorter enzyme version without an apparent signal peptide and secretion of a potentially intracellur enzyme might occur by disintegration of cellular membranes need to be analyzed in further work. In any case, the activation of Lcc8 and Lcc13 here suggested that they might both be induced by ROS signals and used as defense strategies to eliminate oxidative stress as Lcc9.

In our former study, H_2_O_2_-induced oxidative stress-responsive target genes included the gene for the stress-responsive transcription factor Skn7, which is supposed to positively regulate Lcc9 expression (Ko et al. 2009; Liu et al. 2022; Yaakoub et al. 2022). Sequences in the DNA fragments confirmed as binding motifs of Skn7 in ChIP-Seq data of *C. albicans* (Basso et al. 2017) were found in the *lcc9* promoter region (TCTAGA, − 180 to − 174 bp) and also in the promoter regions of *lcc8* (TATGCA, − 334 to − 329 bp to the start codon of the long *lcc8* version) and *lcc13* (TCTAGA, − 162 to − 157 bp), suggesting a possible regulation by Skn7 also of these two genes. However, whether these silent laccase genes are then stress-activated and to what extent they are activated might depend on transcription factors additional to Skn7 induced by different kinds and concentrations of oxygen species (Chen et al. 2008; Quinn et al. 2011; Yaakoub et al. 2022). In comparison with the coculture condition (Liu et al. 2022), exposure to 1 mM H_2_O_2_ might have triggered more or different transcription factors in the *C. cinerea* AIF overexpression transformants resulting in the activation of genes *lcc8* and *lcc13*. Thus, the mechanisms by which oxygen species regulate the transcription of different laccase genes are potentially complex and require further investigation.

**Fig. 9:**
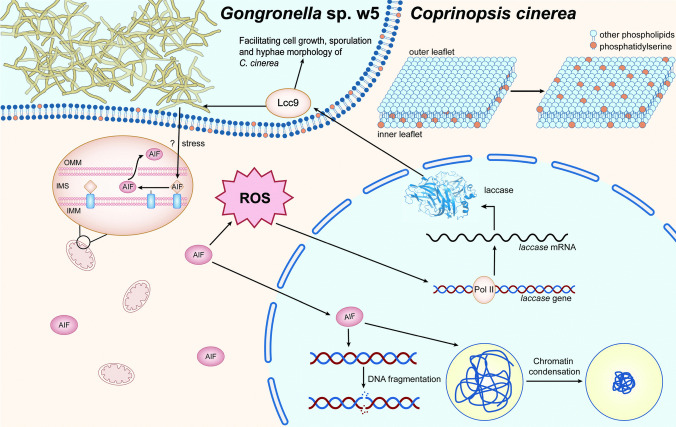
Model for the mechanism of *Cc*AIF1 involving in fungal-fungal interactions. Upon confrontation with *Gongronella* sp. w5, *Cc*AIF1 in *C. cinerea* plays as a ROS regulator and increases the cellular ROS content to trigger the expression of Lcc9 for elimination of the extracellular oxidative stress. Meanwhile, *Cc*AIF1 translocates from mitochondria to cytoplasm to induce apoptotic-like PCD and antagonizes *Gongronella* sp. w5. These strategies mediated by *Cc*AIF1 strengthen cell growth, sporulation and hyphae morphology of *C. cinerea* during cocluture

In summary, we have identified a mitochondrial localized AIF homologue *Cc*AIF1 in the basidiomycete *C. cinerea*. In response to the mucoromycete *Gongronella* sp. w5, *Cc*AIF1 upregulates cellular ROS content of *C. cinerea* to increase Lcc9 expression and translocates to the cytoplasm to involve in apoptotic-like PCD. These comprehensive strategies facilitate *C. cinerea* to confront oxidative stress and enhance mycelial growth during fungal-fungal interactions (Fig. 9). Furthermore for biotechnological applications, our work shows that overexpression of *Cc*AIF1 in combination with appropriate stimulation by oxidative stress is an effective strategy to enhance laccase production in *C. cinerea* axenic culture.

## Supplementary Information

Below is the link to the electronic supplementary material.

Supplementary file1 (PDF 419 KB)

## Data Availability

All data supporting the findings of this study are available within the paper and its Supplementary Information.
