# Supplementary material for: An apoptosis-inducing factor controls programmed cell death and laccase expression during fungal interactions
Source: Appl Microbiol Biotechnol. 2024 Jan 13;108:135. doi: 10.1007/s00253-023-12988-1 (PMC10787690; doi:10.1007/s00253-023-12988-1)
Supplement: Supplementary file 1 [file 253_2023_12988_MOESM1_ESM.pdf]

Applied Microbiology and Biotechnology

An apoptosis-inducing factor controls programmed cell death and laccase expression during fungal interactions

Junnan Fang[1,2,3#], Gang Zhou[1,2,3#], Huifang Zhao[1,2,3], Dengdeng Xie[1,2,3], Jingna Zhang[1,2,3], Ursula Kües[4], Yazhong Xiao[1,2,3], Zemin Fang[1,2,3*], Juanjuan Liu[1,2,3*]

[1] School of Life Sciences, Anhui University, 230601 Hefei, Anhui, China

[2]Anhui Key Laboratory of Modern Biomanufacturing, 230601 Hefei, Anhui, China

[3]Anhui Provincial Engineering Technology Research Center of Microorganisms and Biocatalysis, 230601 Hefei, Anhui, China

[4]Molecular Wood Biotechnology and Technical Mycology, Büsgen - Institute, University of Goettingen, Büsgenweg 2, 37077 Goettingen, Germany

[#] These authors contributed equally.

[*] Corresponding authors

Phone/Fax: +86-551-63861861

Email: zemin_fang@ahu.edu.cn (to ZF), liu_juan825@ ahu.edu.cn (to JL).

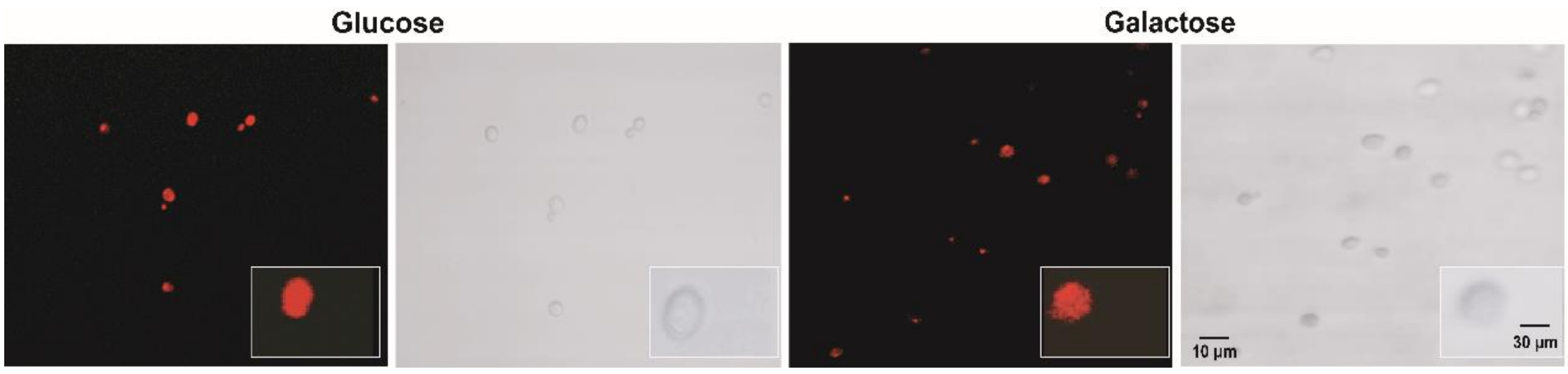

Fig. S1 The comet experiment shows the fragmentation of DNA after *Cc*AIF1 overexpression in yeast. *Ccaif1* overexpression yeast cells were grown on SD-glucose or SD-galactose medium for 3 d. The comet assay was performed for the detection of DNA fragmentation. Scale bars, 10 and 30 µm, respectively.

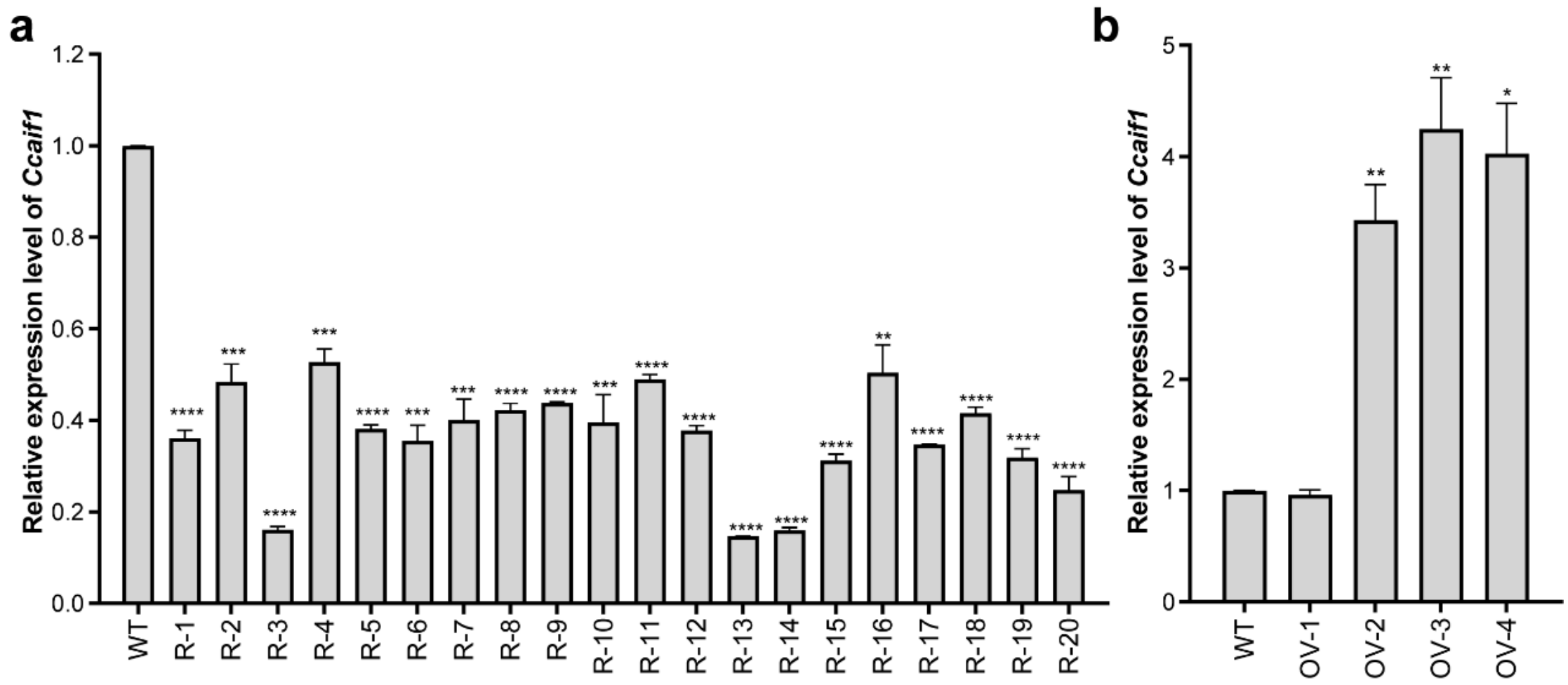

Fig. S2 Transcription level analysis of *Ccaif1* overexpression transformants and silenced transformants. (a) *C. cinerea* protoplasts were transformed with the *Ccaif1* antisense silencing plasmid and analyzed by qRT-PCR, the transcriptional level of *Ccaif1* for twenty potential silencing strains. (b) *C. cinerea* protoplasts were transformed with the *Ccaif1* overexpression plasmid and analyzed by qRT-PCR, the transcriptional level of *Ccaif1* for four potential overexpression strains. *β*-Actin was used as the control. The data were analyzed using a student's *t*-test (**$p < 0.01$, ***$p < 0.001$, ****$p < 0.0001$). Data shown mean ± SD, n = 3.

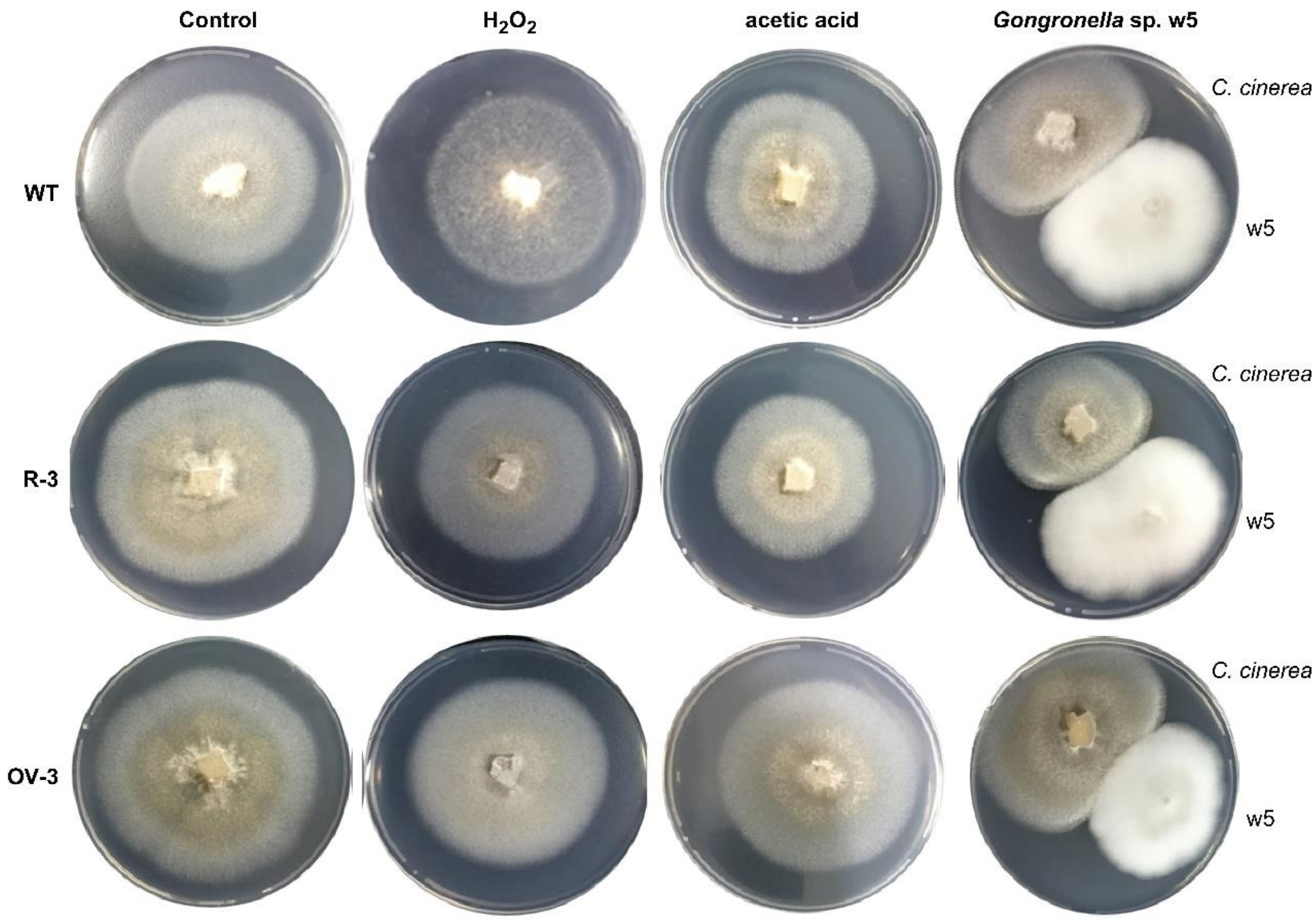

Fig. S3 *Cc*AIF1 is involved in cell growth under oxidative stress. The wild-type strain, *Ccaif1* silencing transformant R-3, and *Ccaif1* overexpression transformant OV-3 were cultured on FAHX agar plates adding 100 mM H_2O_2 or 1 mM acetic acid, or on SAHX agar plates coculturing with *Gongronella* sp. w5.

Table S1 Primers used in this study

Name	Sequence (5'-3')	Purpose
*Cc*AIF-antisense-fwd	CTCCCATCTACACACAACAAGCTTATCGCCGGCAGAAGCCTTGTAAGCAGGGAA	Cloning of *Ccaif1* antisense fragment
*Cc*AIF-antisense-rev	CACTGGCCCTCTGGTCAACTATAATATTATTGTCACCCTCGTTTCTGGATCGGG	Cloning of *Ccaif1* antisense fragment
*Cc*AIF-F	CTCCCATCTACACACAACAAGCTTATCGCCATGTCAGACAAGCGCCAAAACATCG	Cloning of *Ccaif1* overexpression fragment
*Cc*AIF-R	CACTGGCCCTCTGGTCAACTATAATATTATTTAGGCAGAAGCCTTGTAAGCAGG	Cloning of *Ccaif1* overexpression fragment
L22-GFP-F	CTCCCATCTACACACAACAAGCTTATCGCCATGGTGAGCAAGGGCGAGGA	Cloning of *gfp-Ccaif1* overexpression fragment
PF	ACATCCACCATCTCCGTTTTCTCCCAT	PCR of co-transformants
PR	TGACTATAGCAGCCTCCTACCACTG	PCR of co-transformants
qRT-*13048*-F	CTCTGGAGTTATGGTAGGAATGGGC	qRT-PCR of *β-actin*
qRT-*13048*-R	GATGCCATGTTCGATGGGGTACTTG	qRT-PCR of *β-actin*
qRT-*08456*-F	ACAACACGCAAGGGCAAGTCT	qRT-PCR of *Ccaif1*
qRT-*08456*-R	GTCGTCGCCTAAGGACTCTTTGAT	qRT-PCR of *Ccaif1*
qRT-*10894*-F	TTGCTGATTACGTGCCGTGGT	qRT-PCR of *Ccaif2*
qRT-*10894*-R	TTGGATGTACTTTGACATACCCTCGCT	qRT-PCR of *Ccaif2*
qRT-*lcc9*-F	ATGTCCAGGAAACTTTTCTCTCTCG	qRT-PCR of *lcc9*
qRT-*lcc9*-R	ATGTTCGAGACCGTCATGGTACT	qRT-PCR of *lcc9*
CC-F	CCCAAGCTTATGTCAGACAAGCGCCAAAACATCG	Cloning of *Ccaif1* for *S. cerevisiae*
CC-R	CGGAATTCTTAGGCAGAAGCCTTGTAAGCAGGG	Cloning of *Ccaif1* for *S. cerevisiae*
GFP-F	CCCAAGCTTATGGTGAGCAAGGGCGAGGA	Cloning of *GFP-Ccaif1* for *S. cerevisiae*
GFP-linker-R	GATGTTTTGGCGCTTGTCTGACATAGAACCACTACCACTACCTCACTTGTACAGCTCGTCCATGCC	Cloning of *GFP-Ccaif1* for *S. cerevisiae*
CCHP-linker-F	GGCATGGACGAGCTGTACAAGGGTTCAGGTAGTGGTAGTGGTTCTATGTCAGACAAGCGCCAAAACATC	Cloning of *GFP-Ccaif1* for *S. cerevisiae*
CC-R	CGGAATTCTTAGGCAGAAGCCTTGTAAGCAGGG	Cloning of *GFP-Ccaif1* for *S. cerevisiae*

Table S2 Mitotic spores of the wild-type, *Ccaif1* silencing, and *Ccaif1* overexpression *C. cinerea* strains.

Strain name	Absolute number of oidia	Relative number of oidia
**Wild-type**	2.89×10^{8}	1.17×10^{7}
**R-3**	9.77×10^{7}	0.62×10^{7}
**R-13**	9.03×10^{7}	0.59×10^{7}
**R-14**	1.01×10^{8}	0.67×10^{7}
**R-20**	1.52×10^{8}	0.98×10^{7}
**OV-2**	3.67×10^{8}	1.26×10^{7}
**OV-3**	4.26×10^{8}	1.45×10^{7}
**OV-4**	3.93×10^{8}	1.34×10^{7}
